# Mathematical modelling of the COVID-19 pandemic with demographic effects

**DOI:** 10.1186/s42787-021-00118-7

**Published:** 2021-03-17

**Authors:** Abdul A. Kamara, Lagès N. Mouanguissa, Godfrey Okumu Barasa

**Affiliations:** 1 Department of Mathematics and Statistics, Fourah Bay College, University of Sierra Leone, Freetown, Sierra Leone; 2 Department of Mathematics, Ecole Normale Superieure Université Marien Ngouabi, Brazzaville, Congo; 3 Department of Physical Sciences, Jaramogi Oginga Odinga University of Science and Technology, Bondo, Kenya

**Keywords:** Mathematical modelling, COVID-19, Demographic effects, Asymptotic stability

## Abstract

In this paper, a latent infection susceptible–exposed–infectious–recovered model with demographic effects is used to understand the dynamics of the COVID-19 pandemics. We calculate the basic reproduction number (${R}_{0}$) by solving the differential equations of the model and also using next-generation matrix method. We also prove the global stability of the model using the Lyapunov method. We showed that when the ${R}_{0}<1$ or ${R}_{0}\le 1$ and ${R}_{0}>1$ or ${R}_{0}\ge 1$ the disease-free and endemic equilibria asymptotic stability exist theoretically. We provide numerical simulations to demonstrate the detrimental impact of the direct and latent infections for the COVID-19 pandemic.

## Introduction

The COVID-19 is a novel contagious respiratory infection belong to the Coronaviruses family that causes illness ranging from a common cold to severe illness in humans like the Middle East respiratory syndrome (MERS) and severe acute respiratory syndrome (SARS) in adults and children [1, 2]. COVID-19 started in the city of Wuhan, Hubei Province, China, in 2019 and has spread to all parts of the world, affecting more than 200 countries and territories [3]. It is the third coronavirus species to infect human populations in the past two decades [4–6]. As of 25 February 2021, there have been global confirmed cases of over 113 million, and 2.5 million resulted in deaths [3]. Symptoms of the virus are fever, cough, shortness of breath, fatigue, body aches, headache, the loss of taste or smell, sore throat, congestion or runny nose, nausea or vomiting, and diarrhoea [7]. Close contact and respiratory droplets within approximately 6 feet (1.8 m) are the most common primary causes of transmission [8].

According to the World Health Organization (WHO), people who are infected but never developed any symptoms (asymptomatic people) and those who have not yet developed symptom but go on to develop symptoms later (pre-symptomatic people) can also infect others [9]. That is, latent infection is possible and people who contracted the virus can spend between 2 and 14 days before signs and symptoms manifest [7, 10, 11]. Although most of those infected get cured without treatment, there is currently some vaccine and antiviral therapy to prevent contacting the virus. In a mild case, usual flu treatments like antibiotic drugs are used, and in severe cases, supportive treatment like a breathing machine is given to protect vital functions of the organs. The virus infects all ages of humans, but the higher risk is more on adult individuals with severe illness relating to respiratory diseases, organ diseases, and blood diseases [7].

The basic reproduction number $\left({R}_{0}\right)$ is a critical threshold quantity associated with viral transmissibility, and it has been used to understand the transmission of the COVID-19. Epidemiological ${R}_{0}$ is a value used to describe the contagiousness of the pathogen, and it is estimated using incidence data during the first phase of a disease outbreak. It describes the number of people on average that would be infected from a case introduced into a population. The initial COVID-19 pandemic ${R}_{0}$, according to the WHO, was estimated to be between ranges of 1.4 and 2.5 [6]. That is, one infected person will infect an average of 2 persons in his/her lifetime. In the first phase of the epidemic, Zhao et al. [12] estimated the average ${R}_{0}$ for COVID-19, from 3.3 to 5.5, and Read et al. [13] estimated to range between 3.6 and 4.0.

Stability analysis, which has a direct relationship with ${R}_{0}$, is also another way to understand infectious disease. It is believed that when ${R}_{0}$ is above unity, the disease will persist, and the stability is endemic, and when ${R}_{0}$ is less than unity, the disease will die out, and the stability is disease-free. The analysis is done by partitioning the state of individuals in the population into different compartments. For instance, since COVID-19 has an incubation period, the population can be divided into those who are capable of being affected by the virus, call the susceptible (*S*) compartment. When a visibly infected (*I*, i.e. a person confirmed to have the virus) individual is identified, from the S compartment; the infectious person contact and contact–contact form an exposed (*E*) compartment; and those overcoming the illness of the virus and get well form the recovered (*R*) compartment. The SEIR is interpreted using differential equations, where differential equation techniques and simple algebraic methods are used to study the dynamic of the disease. However, the ${R}_{0}$ can also be calculated using the differential equations model at the state when the disease is free from the population (disease-free state).

In this study, a deterministic four-compartment SEIR model is considered to inspect the stability analysis of the COVID-19 pandemic using differential equation techniques. That is, contrary from traditional SEIR model, where an individual in the *E* compartment is infected but not infectious, we consider *E* as another infection transition point but not visibly infectious [14, 15]. This is done by formulating four nonlinear differential equations and provides theoretical and numerical analysis of the model. Our results show that, theoretically, the disease-free and endemic equilibria of the model are locally and globally asymptotically stable and the direct and the rate of infection transmission from an individual after exposure to the virus are detrimental for the COVID-19 pandemic.

## Methodology

### Model framework

In this section, we describe an epidemic transmission SEIR model with demographic changes. The model is used in epidemiology to compute the amount of susceptible, exposed, visibly infected, recovered people in a population (*N*). Since the asymptomatic and pre-symptomatic people can transmit the virus but their symptoms are not visible, they are grouped into the *E* compartment and infection from *E* is referred to as latent infection. This model is used under the following assumptions:

- The population is constant but large.
- The only way a person can leave the susceptible state (*S*) is to become infected either from the exposed (*E*) or from visibly infected (*I*) state or die of natural causes.
- The only way a person can leave the *E* state is to show signs and symptoms of the illness or die of natural death.
- The only way a person can leave the *I* state is to recover from the disease or die from natural death or die as a result of the disease.
- A person who recovered (*R*) from the illness received permanent immunity.
- Age, sex, social status, and race do not affect the probability of being infected.
- The member of the population has the same contacts with one another equally.
- All births are into the susceptible state, and it is assumed that the birth and natural death rates are equal.

**Fig. 1:**
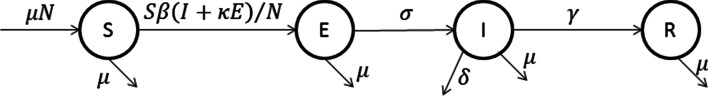
The latent infection SEIR model flow diagram

The transmission is measured at $S\beta \left(I+\kappa E\right)/N$, where $\beta$ is the direct transmission rate, and $\kappa$ is the proportional rate constant when an uninfected individual comes into contact with an individual from state *E*. We assume natural birth and death rate to be measured at an equal rate $\mu$ and induced death rate measured at $\delta$. The rate for an individual to move from state *E* to state *I* is measured at rate $\sigma ,$, and the rate of recovery is measured at $\gamma$. Figure 1 represents the latent infection *SEIR* model, which is described using the system of nonlinear ordinary differential equations

1 $$\begin{aligned}&\frac{\mathrm{d}S\left(t\right)}{\mathrm{d}t}=\mu N-\beta \frac{S\left(I+\kappa E\right)}{N}-\mu S,\\ &\frac{\mathrm{d}E\left(t\right)}{\mathrm{d}t}=\beta \frac{S\left(I+\kappa E\right)}{N}-\left(\mu +\sigma \right)E, \\ &\frac{\mathrm{d}I\left(t\right)}{\mathrm{d}t}=\sigma E-\left(\mu +\gamma +\delta \right)I,\\ &\frac{\mathrm{d}R(t)}{\mathrm{d}t}=\gamma I-\mu R,\end{aligned}$$

where $S\left(t\right)=S,E\left(t\right)=E,I\left(t\right)=I$ and $R\left(t\right)=R$ denote the number of susceptible, exposed, infectious, and remove individuals at time $t$, respectively, and $N=S+E+I+R$. System (1) is subjected to the initial condition

2 $$S\left(0\right)\ge 0, E\left(0\right)\ge 0, I\left(0\right)\ge 0,\mathrm{and} R\left(0\right)\ge 0$$

For simplicity system (1) is reduced to a proportional framework given as

3 $$\begin{aligned}&\frac{\mathrm{d}s\left(t\right)}{\mathrm{d}t}=\mu -\beta s\left(i+\kappa e\right)-\mu s,\\ &\frac{\mathrm{d}e\left(t\right)}{\mathrm{d}t}=\beta s\left(i+\kappa e\right)-\left(\mu +\sigma \right)e,\\ &\frac{\mathrm{d}i\left(t\right)}{\mathrm{d}t}=\sigma e-\left(\mu +\gamma +\delta \right)i,\\ &\frac{\mathrm{d}r(t)}{\mathrm{d}t}=\gamma i-\mu r, \end{aligned}$$

where $s=S/N,e=E/N,i=I/N$, and $r=r/N$. By considering the total population

$$s+e+i+r=1 \Rightarrow r=1-s-e-i,$$

therefore, system (3) can be reduced to

4 $$\begin{aligned}&\frac{\mathrm{d}s(t)}{\mathrm{d}t}=\mu -\beta s\left(i+\kappa e\right)-\mu s,\\ &\frac{\mathrm{d}e(t)}{\mathrm{d}t}=\beta s\left(i+\kappa e\right)-\left(\mu +\sigma \right)e,\\ &\frac{\mathrm{d}i(t)}{\mathrm{d}t}=\sigma e-\left(\mu +\gamma +\delta \right)i. \end{aligned}$$

### Positivity of the solution

Assume that system (4) has a global solution corresponding to non-negative initial conditions. Then, the following Lemma confirms that the solution is non-negative at all times.

#### Lemma 1

*If s*(0) ≥ 0*, e*(0) ≥ 0 *and i*(0) ≥ 0 *then the solution s*(*t*)*, e*(*t*) *and i*(*t) are all positive for all *$t\ge 0$.

#### ***Proof***.

We use the contradiction: we assuming there exists positive real ${t}_{1},{t}_{2}$ and ${t}_{3}$ for which one of the conditions hold:

1. $s\left({t}_{1}\right)=0, \mathrm{d}s\left({t}_{1}\right)/\mathrm{d}t<0$, and for all $0\le t\le {t}_{1}$ one has that $e\left(t\right)\ge 0$ and $i\left(t\right)\ge 0$;
2. $e\left({t}_{2}\right)=0, \mathrm{d}e\left({t}_{2}\right)/\mathrm{d}t<0$, and for all $0\le t\le {t}_{2}$ one has that $s\left(t\right)\ge 0$ and $i\left(t\right)\ge 0$;
3. $i\left({t}_{3}\right)=0, \mathrm{d}i\left({t}_{3}\right)/\mathrm{d}t<0$, and for all $0\le t\le {t}_{3}$ one has that $s\left(t\right)\ge 0$ and $e\left(t\right)\ge 0$.

Condition (I) contradicts if $s\left(t\right)\ge 0$, $\mathrm{d}s\left({t}_{1}\right)/\mathrm{d}t=\mu >0$. Also, condition (II) contradicts because $e\left(t\right)\ge 0$, $\mathrm{d}e\left({t}_{2}\right)/\mathrm{d}t=\beta si\ge 0$. Finally, condition (III) contradicts since for $i\left(t\right)\ge 0$, $\mathrm{d}i\left({t}_{3}\right)/\mathrm{d}t=\sigma E\ge 0.$. Thus, the solutions of $s(t), e(t) and i(t)$ remain positive for all $t>0$.

Hence, the positively invariant for the system (4) is

5 $$\varOmega =\left\{s\left(t\right), e\left(t\right), i\left(t\right)\epsilon {R}_{+}^{3},s\left(t\right)+e\left(t\right)+ i\left(t\right)\le 1\right\}.$$

$$\square$$

### The equilibrium points and reproduction number calculations of the model

There are two equilibrium points for the system (4), i.e. the disease-free equilibrium (DFE), the state when the disease is absent, and the endemic equilibrium (EE), which is the state when the disease continues to persist in the population.

Let the DFE points of the model are denoted as ${E}^{0}=\left({s}^{0},{e}^{0},{i}^{0}\right)$ and represent a system (4) at ${E}^{0}$ as

6 $$\begin{aligned} &\mu -\beta {s}^{0}\left({i}^{0}+\kappa {e}^{0}\right)-\mu {s}^{0}=0,\\ & \beta {s}^{0}\left({i}^{0}+\kappa {e}^{0}\right)-\left(\mu +\sigma \right){e}^{0}=0,\\ &\sigma {e}^{0}-\left(\mu +\gamma +\delta \right){i}^{0}=0. \end{aligned}$$

In terms of ${i}^{0}$, from the last equation of (6), we get

$${e}^{0}=\frac{\left(\mu +\gamma +\delta \right){i}^{0}}{\sigma }.$$

Adding the first two equations of (6) and substituting for ${e}^{0}$, we get

$${s}^{0}=1-\frac{\left(\mu +\sigma \right)\left(\mu +\gamma +\delta \right){i}^{0}}{\mu \sigma }.$$

Because at the disease-free state no one has the infection then, ${i}^{0}={e}^{0}=0$. We can see that ${E}^{0}=\left({s}^{0},{e}^{0},{i}^{0}\right)=\left(\mathrm{1,0},0\right).$

Also, the EE points are denoted as${E}^{*}=\left({s}^{*},{e}^{*},{i}^{*}\right)$, where ${s}^{*},{e}^{*}$ and ${i}^{*}$ are calculated by letting ${s}^{0}={s}^{*} , {e}^{0}={e}^{*} , {i}^{0}={i}^{*}$, and then, the second equation of (6) becomes

7 $$\begin{aligned} &\beta \left(1+\frac{\kappa \left(\mu +\gamma +\delta \right)}{\sigma }\right)-\beta \left(\frac{\left(\mu +\sigma \right)\left(\mu +\gamma +\delta \right)}{\mu \sigma }\right)\left(1+\frac{\kappa \left(\mu +\gamma +\delta \right)}{\sigma }\right){i}^{*}\\ & \quad -\,\frac{\left(\mu +\sigma \right)\left(\mu +\gamma +\delta \right)}{\sigma }=0. \end{aligned}$$

Multiplying both sides of (7) by $\sigma /\left(\mu +\sigma \right)\left(\mu +\gamma +\delta \right)$, we get

$$\frac{\sigma \beta }{\left(\mu +\sigma \right)\left(\mu +\gamma +\delta \right)}\left(1+\frac{\kappa \left(\mu +\gamma +\delta \right)}{\sigma }\right)-\frac{\beta }{\mu }\left(1+\frac{\kappa \left(\mu +\gamma +\delta \right)}{\sigma }\right){i}^{*}-1=0,$$

which implies that

$$\frac{\beta }{\mu }\left(1+\frac{\kappa \left(\mu +\gamma +\delta \right)}{\sigma }\right){i}^{*}=\frac{\sigma \beta }{\left(\mu +\sigma \right)\left(\mu +\gamma +\delta \right)}\left(1+\frac{\kappa \left(\mu +\gamma +\delta \right)}{\sigma }\right)-1,$$

Hence

$${i}^{*}=\frac{\mu }{\beta }\left(\frac{\sigma }{\sigma +\kappa \left(\mu +\gamma +\delta \right)}\right)\left(\frac{\sigma \beta }{\left(\mu +\sigma \right)\left(\mu +\gamma +\delta \right)}\left(1+\frac{\kappa \left(\mu +\gamma +\delta \right)}{\sigma }\right)-1\right),$$

also

$$\begin{aligned} &{e}^{*}=\left(\frac{\left(\mu +\gamma +\delta \right)}{\sigma }\right){i}^{*},\\ &{s}^{*}=1-\frac{\left(\mu +\sigma \right)\left(\mu +\gamma +\delta \right){i}^{*}}{\mu \sigma }. \end{aligned}$$

To understand the stability of the model, we need an expression to estimate the basic reproduction number ($${R}_{0})$$.

However, from (6) let${s}^{0}=s , {e}^{0}=e , {i}^{0}=i$, in terms of $i$ the second equation of (6) becomes

8 $$\beta i\left(1+\frac{\kappa \left(\mu +\gamma +\delta \right)}{\sigma }\right)\left(1-\frac{\left(\mu +\sigma \right)\left(\mu +\gamma +\delta \right)i}{\mu \sigma }\right)-\frac{\left(\mu +\sigma \right)\left(\mu +\gamma +\delta \right)i}{\sigma }=0.$$

By the factorising method, we get

9 $$i\left(\beta \left(1+\frac{\kappa \left(\mu +\gamma +\delta \right)}{\sigma }\right)\left(1-\frac{\left(\mu +\sigma \right)\left(\mu +\gamma +\delta \right)i}{\mu \sigma }\right)-\frac{\left(\mu +\sigma \right)\left(\mu +\gamma +\delta \right)}{\sigma }\right)=0,$$

either $$i=0$$ or

$$\begin{aligned}&\beta \left(1+\frac{\kappa \left(\mu +\gamma +\delta \right)}{\sigma }\right)-\beta \left(\frac{\left(\mu +\sigma \right)\left(\mu +\gamma +\delta \right)}{\mu \sigma }\right)\left(1+\frac{\kappa \left(\mu +\gamma +\delta \right)}{\sigma }\right)i-\frac{\left(\mu +\sigma \right)\left(\mu +\gamma +\delta \right)}{\sigma }=0 , \\ &i=\left(\frac{{\mu \sigma }^{2}}{\left(\mu +\sigma \right)\left(\mu +\gamma +\delta \right)\left(\sigma +\kappa \left(\mu +\gamma +\delta \right)\right)}\right)\left[\left(1+\frac{\kappa \left(\mu +\gamma +\delta \right)}{\sigma }\right)-\frac{\left(\mu +\sigma \right)\left(\mu +\gamma +\delta \right)}{\beta \sigma }\right], \end{aligned}$$

Equating the highest $i$ value from (9) to zero, we get

10 $$\frac{\sigma \beta }{\left(\mu +\sigma \right)\left(\mu +\gamma +\delta \right)}\left(1+\frac{\kappa \left(\mu +\gamma +\delta \right)}{\sigma }\right)=1.$$

Since the threshold for ${R}_{0}$ is unity, we then assume

11 $${R}_{0}=\frac{\sigma \beta }{\left(\mu +\sigma \right)\left(\mu +\gamma +\delta \right)}\left(1+\frac{\kappa \left(\mu +\gamma +\delta \right)}{\sigma }\right).$$

Equation (11) is justified using the next-generation matrix method defined in [16] as $K=\rho (F{\mathcal{V}}^{-1})$, where $\rho (F{\mathcal{V}}^{-1})$ is the spectral radius of the matrix $F{\mathcal{V}}^{-1}$ and the largest eigenvalue of $K$ is the ${R}_{0}$. $F$ and $\mathcal{V}$ are the matrices associated with the DFE points defined as

$$\begin{aligned}&F=\left(\begin{array}{cc}\kappa \beta & \beta \\ 0& 0\end{array}\right) \mathrm{and}\mathcal{V}=\left(\begin{array}{cc}\mu +\sigma & 0\\ -\sigma & \mu +\delta +\gamma \end{array}\right),\\ & K=\left(\begin{array}{cc}\kappa \beta & \beta \\ 0& 0\end{array}\right)\left(\begin{array}{cc}\frac{1}{\mu +\sigma }& 0\\ \frac{\sigma }{\left(\mu +\sigma \right)\left(\mu +\gamma +\delta \right)}& \frac{1}{\left(\mu +\gamma +\delta \right)}\end{array}\right)\\ &K=\left(\begin{array}{cc}\frac{\beta }{\mu +\sigma }\left(\kappa +\frac{\sigma }{\left(\mu +\gamma +\delta \right)}\right)& \frac{\beta }{\left(\mu +\gamma +\delta \right)}\\ 0& 0\end{array}\right) \end{aligned}$$

The largest eigenvalues of ${\rm K}$ is as (11) given as

$${R}_{0}=\frac{\sigma \beta }{\left(\mu +\sigma \right)\left(\mu +\gamma +\delta \right)}\left(1+\frac{\kappa \left(\mu +\gamma +\delta \right)}{\sigma }\right).$$

Hence

$${s}^{*}=\frac{1}{{R}_{0}}, {e}^{*}=\frac{\mu \left({R}_{0}-1\right)}{{R}_{0}\left(\mu +\sigma \right)}, {i}^{*}=\frac{\mu \sigma \left({R}_{0}-1\right)}{{R}_{0}\left(\mu +\sigma \right)\left(\mu +\gamma +\delta \right)}.$$

### Stability analysis of the disease-free equilibrium points

#### Theorem 1

*If* ${R}_{0}<1$ *and* $\kappa \beta <\left(\mu +\sigma \right)+\left(2\mu +\gamma +\delta +\sigma \right)$ *, then the DFE is locally asymptotically stable in* $\varOmega$.

#### *Proof*

The Jacobian matrix of system (3) associated with DFE is given as

12 $${J}_{\left(\mathrm{1,0},0\right)}=\left(\begin{array}{ccc}-\mu & -\kappa \beta & -\beta \\ 0& \kappa \beta -\left(\mu +\sigma \right)& \beta \\ 0& \sigma & -\left(\mu +\gamma +\delta \right)\end{array}\right),$$

with characteristic polynomial

$$P\left(\leftthreetimes \right)=\left(\mu +\leftthreetimes \right)\left[{\leftthreetimes }^{2}+\left(\left(\mu +\sigma \right)-\kappa \beta +\left(\mu +\gamma +\delta \right)\right)\leftthreetimes +\left(\left(\mu +\sigma \right)-\kappa \beta \right)\left(\mu +\gamma +\delta \right)-\sigma \beta \right],$$

where $\leftthreetimes$ is an eigenvalue. It is easy to see that for Theorem 1 to satisfy

$$\left(\mu +\sigma \right)-\kappa \beta +\left(\mu +\gamma +\delta \right)>0\Rightarrow \kappa \beta <\left(\mu +\sigma \right)+\left(2\mu +\gamma +\delta +\sigma \right),$$

and

$$\left(\left(\mu +\sigma \right)-\kappa \beta \right)\left(\mu +\gamma +\delta \right)-\sigma \beta >0\Rightarrow {R}_{0}=\frac{\beta }{\left(\mu +\sigma \right)}\left(\frac{\sigma }{\left(\mu +\gamma +\delta \right)}+\kappa \right)<1.$$

The proof of Theorem 1 is complete.

#### Theorem 2

*If* ${R}_{0}\le 1$, *the DFE is globally asymptotically stable in* $\varOmega$.

#### *Proof*

To prove the global asymptotic stability (GAS) of the DFE, we construct the following Lyapunov function $V{:}\,\varOmega \to R$, where $V\left(s,e,i\right)=i\left(t\right)$. Then, the time derivative of $V$ is given as

$$\frac{\mathrm{d}V}{\mathrm{d}t}=\frac{\mathrm{d}{i}^{0}}{\mathrm{d}t}=\frac{\mathrm{d}{e}^{0}}{\mathrm{d}t},$$

since at the equilibrium points $\mathrm{d}{i}^{0}/\mathrm{d}t=\mathrm{d}{e}^{0}/\mathrm{d}t=0$. Therefore

13 $$\frac{\mathrm{d}V}{\mathrm{d}t}=\beta {s}^{0}\left({i}^{0}+\kappa {e}^{0}\right)-\left(\mu +\sigma \right){e}^{0}.$$

Substituting ${s}^{0}$ and ${e}^{0}$ into (13), we get

14 $$\begin{aligned} & \frac{\mathrm{d}V}{\mathrm{d}t}=\beta \left(1+\frac{\kappa \left(\mu +\gamma +\delta \right)}{\sigma }\right){i}^{0}-\beta \left(\frac{\left(\mu +\sigma \right)\left(\mu +\gamma +\delta \right)}{\mu \sigma }\right)\left(1+\frac{\kappa \left(\mu +\gamma +\delta \right)}{\sigma }\right){\left({i}^{0}\right)}^{2}\\ &\quad -\,\frac{\left(\mu +\sigma \right)\left(\mu +\gamma +\delta \right){i}^{0}}{\sigma } , \end{aligned}$$

By the factorisation method

$$\begin{aligned}&\frac{\mathrm{d}V}{\mathrm{d}t}=\frac{\left(\mu +\sigma \right)\left(\mu +\gamma +\delta \right){i}^{0}}{\sigma }\left[\frac{\sigma \beta }{\left(\mu +\sigma \right)\left(\mu +\gamma +\delta \right)}\left(1+\frac{\kappa \left(\mu +\gamma +\delta \right)}{\sigma }\right)\right.\\& \quad \left.- \frac{\left(\mu +\sigma \right)\left(\mu +\gamma +\delta \right)}{\mu \sigma }\left(\frac{\sigma \beta }{\left(\mu +\sigma \right)\left(\mu +\gamma +\delta \right)}\left(1+\frac{\kappa \left(\mu +\gamma +\delta \right)}{\sigma }\right)\right){i}^{0}-1\right].\end{aligned}$$

Substituting ${R}_{0}$, we get

15 $$\frac{\mathrm{d}V}{\mathrm{d}t}=\frac{\left(\mu +\sigma \right)\left(\mu +\gamma +\delta \right){i}^{0}}{\sigma }\left[{R}_{0}-\frac{\left(\mu +\sigma \right)\left(\mu +\gamma +\delta \right){R}_{0}}{\mu \sigma }{i}^{0}-1\right].$$

Thus, $\mathrm{d}V/\mathrm{d}t\le 0$ for ${R}_{0}\le 1$. Furthermore, if ${R}_{0}<1$ then $\mathrm{d}V/\mathrm{d}t=0\iff {i}^{0}=0$ and if ${R}_{0}=1$ then $\mathrm{d}V/\mathrm{d}t=0$. Hence, by Lasalle invariance principle [17], the DFE point is GAS.

### Stability analysis of the endemic equilibrium

#### Theorem 3

*If* ${R}_{0}>1$ *, the endemic equilibrium is locally asymptotically stable*.

#### *Proof*

To prove the LAS of the endemic equilibrium, we consider the Jacobian matrix associated with ${E}^{*}$, that is

16 $${J}_{{E}^{*}}=\left(\begin{array}{ccc}-\mu -\beta \left({i}^{*}+\kappa {e}^{*}\right)& -\kappa \beta {s}^{*}& -\beta {s}^{*}\\ \beta \left({i}^{*}+\kappa {e}^{*}\right)& \kappa \beta {s}^{*}-\left(\mu +\sigma \right)& \beta {s}^{*}\\ 0& \sigma & -\left(\mu +\gamma +\delta \right)\end{array}\right),$$

Substituting for ${s}^{*},{e}^{*}$ and ${i}^{*}$, we get

17 $${J}_{{E}^{*}}=\left(\begin{array}{ccc}\mu {R}_{0}& -\frac{\kappa \beta }{{R}_{0}}& -\frac{\beta }{{R}_{0}}\\ \mu \left({R}_{0}-1\right)& \frac{\kappa \beta }{{R}_{0}}-\left(\mu +\sigma \right)& \frac{\beta }{{R}_{0}}\\ 0& \sigma & -\left(\mu +\gamma +\delta \right)\end{array}\right),$$

if $\lambda$ is an eigenvalue, then

$${J}_{{E}^{*}}-\lambda {\varvec{I}}=\left(\begin{array}{ccc}\mu {R}_{0}-\lambda & -\frac{\kappa \beta }{{R}_{0}}& -\frac{\beta }{{R}_{0}}\\ \mu \left({R}_{0}-1\right)& \frac{\kappa \beta }{{R}_{0}}-\left(\mu +\sigma \right)-\lambda & \frac{\beta }{{R}_{0}}\\ 0& \sigma & -\left(\mu +\gamma +\delta \right)-\lambda \end{array}\right),$$

where ${\varvec{I}}$ is a three-dimensional identity matrix which by matrix simplification method we then get

$$P\left(\lambda \right)={\lambda }^{3}+a{\lambda }^{2}+b\lambda +c,$$

where

$$a=\mu {R}_{0}+\left(\mu +\gamma +\delta \right)+\left(\mu +\sigma \right)\left(1-\frac{\kappa \beta }{{\left(\mu +\sigma \right)R}_{0}}\right).$$

From ${R}_{0}$, we get

18 $$\frac{\sigma \beta }{{\left(\mu +\gamma +\delta \right)\left(\mu +\sigma \right)R}_{0}}=1-\frac{\kappa \beta }{\left(\mu +\sigma \right){R}_{0}},$$

hence

$$\begin{aligned}&a=\mu {R}_{0}+\left(\mu +\gamma +\delta \right)+\frac{\sigma \beta }{{\left(\mu +\gamma +\delta \right)R}_{0}}>0,\\ &b=\mu {R}_{0}\left(\mu +\gamma +\delta \right)+\mu {R}_{0}\left(\mu +\sigma \right)+\left(\mu +\gamma +\delta \right)\left(\mu +\sigma \right)-\mu \kappa \beta -\frac{\kappa \beta \left(\mu +\gamma +\delta \right)}{{R}_{0}}-\frac{\kappa \beta }{{R}_{0}},\\ &b=\mu {R}_{0}\left(\mu +\gamma +\delta \right)+\mu {R}_{0}\left(\mu +\sigma \right)\left(1-\frac{\kappa \beta }{\left(\mu +\sigma \right){R}_{0}}\right),\end{aligned}$$

using (18) we get

$$\begin{aligned}&b=\mu {R}_{0}\left(\mu +\gamma +\delta \right)+\frac{\mu \beta }{\left(\mu +\gamma +\delta \right)}>0.\\ &c=\frac{\mu \kappa \beta \left({R}_{0}-1\right)}{{R}_{0}}-\mu \kappa \beta \left(\mu +\gamma +\delta \right)+\mu \left(\mu +\sigma \right)\left(\mu +\gamma +\delta \right){R}_{0}-\mu \kappa \beta ,\\ &c=\frac{\mu \kappa \beta \left({R}_{0}-1\right)}{{R}_{0}}>0,\end{aligned}$$

and

$$ab-c=\frac{\mu \kappa \beta }{{R}_{0}}\left(\frac{\beta }{\left(\mu +\gamma +\delta \right)}+1\right)+\mu \beta \left(\frac{\mu {R}_{0}}{\left(\mu +\gamma +\delta \right)}+1\right)+\mu {R}_{0}\left(\mu +\gamma +\delta \right)\left(\mu {R}_{0}+\mu +\gamma +\delta \right).$$

Since $a>0,b>0,c>0,$ and $ab-c>0$, according to the Routh–Hurwitz criterion, the endemic equilibrium of system (4) is LAS.

#### Theorem 4

*The endemic equilibrium point is globally asymptotically stable on* $\varOmega$.

#### *Proof*

We construct the following Lyapunov function ${V}_{1}: {\varOmega }_{+}\to R$, where ${\varOmega }_{+}=\left\{s\left(t\right),e\left(t\right),i\left(t\right)\in\varOmega /s\left(t\right)>0,e\left(t\right)>0,i\left(t\right)>0\right\}$ given by

19 $${V}_{1}\left(X,t\right)=\frac{1}{2}\left({X}_{1}^{2}+{X}_{2}^{2}+{X}_{3}^{2}\right),$$

${X}_{1}=s-{s}^{*} , {X}_{2}=e-{e}^{*}$, and${X}_{3}=i-{i}^{*}$,${L}_{1}$. We can see that ${V}_{1}\left(X,t\right)>0$ and ${V}_{1}\left(\mathrm{0,0},0\right)=\left(\mathrm{0,0},0\right)$ for all $\left({X}_{1}, {X}_{2},{X}_{3}\right)$ in the region, that makes ${V}_{1}$ positive definite*.* We need to verify that $\mathrm{d}{V}_{1}/\mathrm{d}t\le 0$ (negative definite)*.* The time derivative of ${\mathrm{V}}_{1}$ is

$$\frac{\mathrm{d}{V}_{1}}{\mathrm{d}t}={X}_{1}\frac{\mathrm{d}{X}_{1}}{\mathrm{d}t}+{X}_{2}\frac{\mathrm{d}{X}_{2}}{\mathrm{d}t}+{X}_{3}\frac{\mathrm{d}{X}_{3}}{\mathrm{d}t},$$

where $\mathrm{d}{X}_{1}/\mathrm{d}t=\mathrm{d}s/\mathrm{d}t, \mathrm{d}{X}_{2}/\mathrm{d}t=\mathrm{d}(e)/\mathrm{d}t$ and $\mathrm{d}{X}_{3}/\mathrm{d}t=\mathrm{d}(i)/\mathrm{d}t.$ Hence,

$$\frac{\mathrm{d}{V}_{1}}{\mathrm{d}t}={X}_{1}\left(\mu -\beta s\left(i+\kappa e\right)-\mu s\right)+{X}_{2}\left(\beta s\left(i+\kappa e\right)-\left(\mu +\sigma \right)e\right)+{X}_{3}\left(\sigma e-\left(\mu +\gamma +\delta \right)i\right).$$

From

$$\begin{aligned}&\mu -\beta {s}^{*}\left({i}^{*}+\kappa {e}^{*}\right)-\mu {s}^{*}=0,\\ &\beta {s}^{*}\left({i}^{*}+\kappa {e}^{*}\right)-\left(\mu +\sigma \right){e}^{*}=0,\\ & \sigma {e}^{*}-\left(\mu +\gamma +\delta \right){i}^{*}=0, \\ &{s}^{*}=\frac{\mu }{\left\{\mu +\beta \left({i}^{*}+\kappa {e}^{*}\right)\right\}}, {e}^{*}=\frac{\beta {s}^{*}\left({i}^{*}+\kappa {e}^{*}\right)}{\left(\mu +\sigma \right)}, {i}^{*}=\frac{\sigma {e}^{*}}{\left(\mu +\gamma +\delta \right)},\end{aligned}$$

Therefore,

$${X}_{1}=s-\frac{\mu }{\left\{\mu +\beta \left({i}^{*}+\kappa {e}^{*}\right)\right\}} , {X}_{2}=e-\frac{\beta {s}^{*}\left({i}^{*}+\kappa {e}^{*}\right)}{\left(\mu +\sigma \right)}, \mathrm{and}\, {X}_{3}=i-\frac{\sigma {e}^{*}}{\left(\mu +\gamma +\delta \right)},$$

Hence

$$\begin{aligned}&\frac{\mathrm{d}{V}_{1}}{\mathrm{d}t}=\left(s-\frac{\mu }{\left\{\mu +\beta \left({i}^{*}+\kappa {e}^{*}\right)\right\}}\right)\left[\mu -\beta s\left(i+\kappa e\right)-\mu s\right]+\left(i-\frac{\sigma {e}^{*}}{\left(\mu +\gamma +\delta \right)}\right)\left[\sigma e-\left(\mu +\gamma +\delta \right)i\right] \\ &\quad +\,\left(e-\frac{\beta {s}^{*}\left({i}^{*}+\kappa {e}^{*}\right)}{\left(\mu +\sigma \right)}\right)\left[\beta s\left(i+\kappa e\right)-\left(\mu +\sigma \right)e\right], \end{aligned}$$

If we assume$s={s}^{*} , e={e}^{*}$, and$i={i}^{*}$, we get

$$\begin{aligned} &\frac{\mathrm{d}{V}_{1}}{\mathrm{d}t}=-\left\{\mu +\beta \left({i}^{*}+\kappa {e}^{*}\right)\right\}{\left({s}^{*}-\frac{\mu }{\left\{\mu +\beta \left({i}^{*}+\kappa {e}^{*}\right)\right\}}\right)}^{2}-\left(\mu +\sigma \right){\left({e}^{*}-\frac{\beta {s}^{*}\left({i}^{*}+\kappa {e}^{*}\right)}{\left(\mu +\sigma \right)}\right)}^{2} \\ &\quad -\,\left(\mu +\gamma +\delta \right){\left({i}^{*}-\frac{\sigma {e}^{*}}{\left(\mu +\gamma +\delta \right)}\right)}^{2}\le 0 , \end{aligned}$$

which conclude the proof of Theorem 4.

## Results and discussion

In this section, we illustrate the DFE and EE theorems numerically using the integration technique in R-software. The model parameter values are obtained from COVID-19 literature [3, 15–17], and we focus our analysis in a small settlement approximately 1000 population. As of 10 June 2020, the global case fatality rate was estimated as the ratio of total deaths and total confirmed cases $\left(\delta =\mathrm{408,025}/\mathrm{7,145,539}=0.057\right)$ [3], the incubation period has a mean average of 5.2 days and the recovery period is 5.8 days [18], i.e. $\left(\sigma =1/5.2=0.192, \gamma =1/5.8=0.172\right)$. The birth and death rate is assumed to be $\left(\mu =0.05\right)$, the proportion of latent infection rate ($\kappa )=0.5$ [19] and $\beta =0.533$ as in [20].

**Fig. 2:**
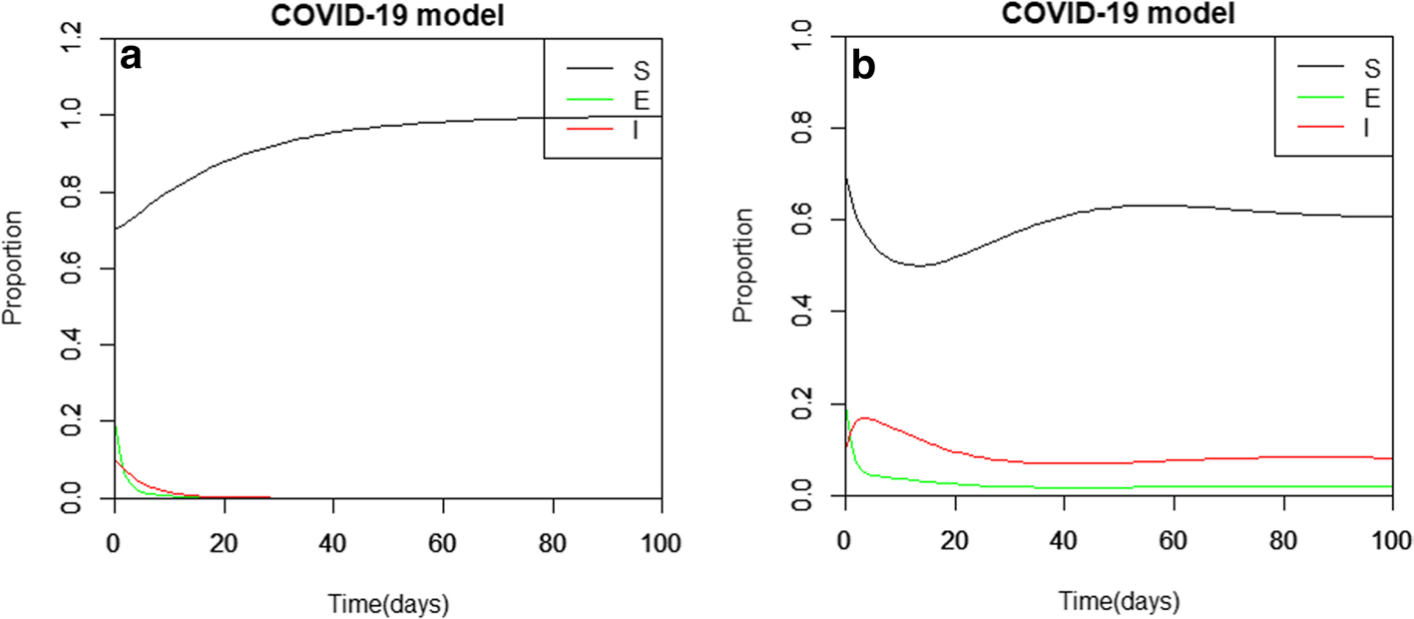
The latent infection SEIR model asymptotic stability analyses

Firstly, we investigate the DFE by assuming $\beta =0.0533$; we observe that when ${R}_{0}=0.163$ in Fig. 2a, Theorems 1 and 3 are satisfied for the DFE to be asymptotically stable. It is observed that when states *E* and *I* are decreasing, the susceptible population approaches unity with increasing time. Also, in Fig. 2b, we observe that when $\beta =0.533$, and ${R}_{0}=1.63$, Theorems 2 and 4 for the EE of the model to be asymptotically stable are satisfied. It is observed that increasing the ***I*** proportion the ***S*** proportion declines until at a certain point in time when the ***I*** proportion started to decrease and the **S** proportion then increases. The decrease in the trajectories in the case of the ***E*** state is the result of the increase in asymptomatic individual to the visibly infectious state and natural death, whereas for the *I* state is the results of the increase in the recovered individual and those who might have died of natural or virus death.

**Fig. 3:**
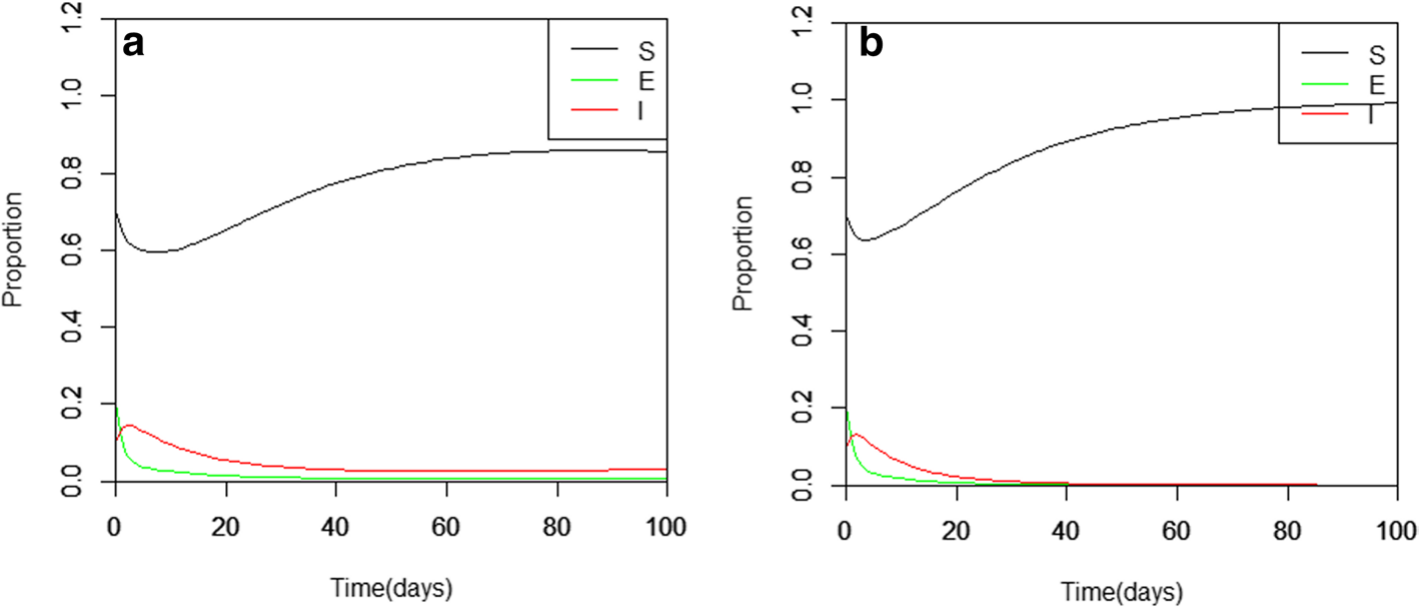
The latent infection SEIR model effects, where ${\varvec{\kappa}}=0.3$ (**a**) and ${\varvec{\kappa}}=0.1$ (**b**)

From a mathematical point of view, it is easy to see that the EE tends to DFE which is dependent on the decreasing rate of $\kappa$ and $\beta$. We investigate the effect of the direct and latent infection rate numerically by keeping the EE parameter values constant and regulating the degree of $\kappa$ and $\beta$. That is, the positive effect for the direct and latent infection parameters is noticeable when the magnitude of $\kappa$ and $\beta$ decreases, and their output is similar to that of Fig. 2a. It is observed in Fig. 3 that as $\kappa$ decreases the endemic trajectory patterns are similar to the DFE curves in Fig. 2a.

**Fig. 4:**
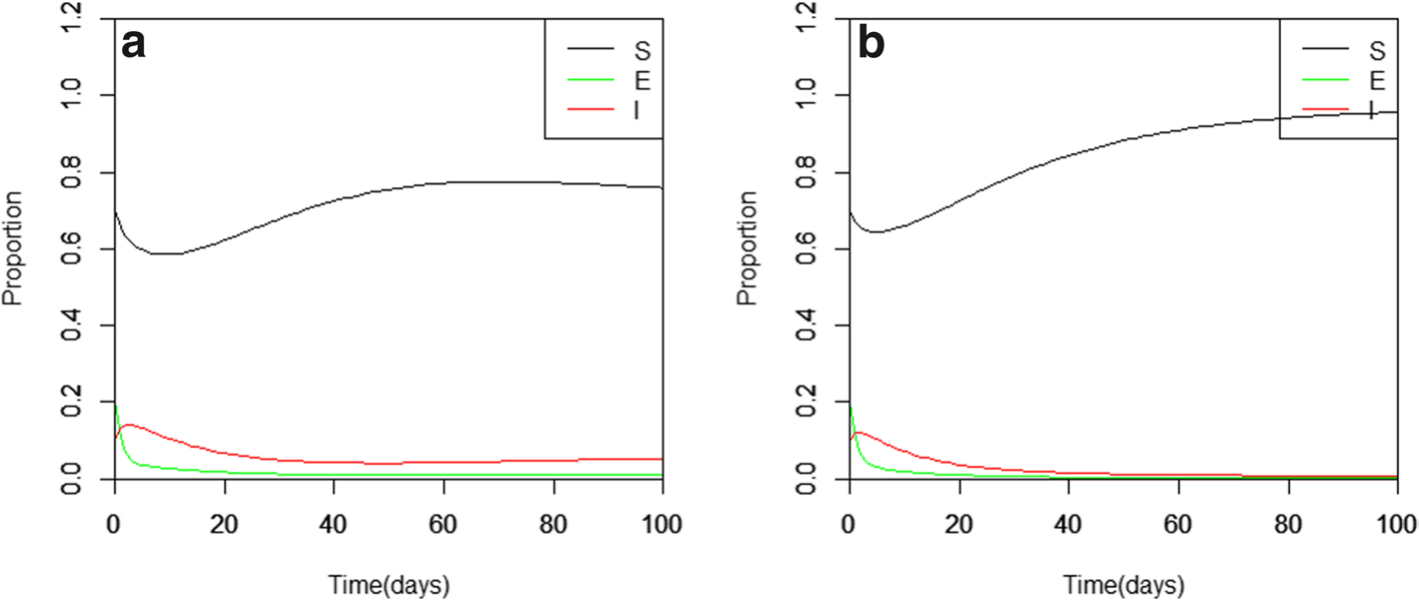
The latent infection SEIR model with direct transmission effects where ${\varvec{\beta}}=0.433$ (**a**) and ${\varvec{\beta}}=0.333$ (**b**)

Also, we observe that when $\beta$ is lower in magnitude, lesser susceptible individuals become infected as the curve tends to increase in proportion. In Figs. 3 and 4, it is easy to see that direct and latent infection transmissions can enhance the persistence of the COVID-19 pandemic.

## Conclusion

In this paper, we formulate a latent infection SEIR model to investigate the stability analysis of the COVID-19 pandemic with demographic effects. We use differential equation techniques and simple algebraic procedures to describe the dynamics of the model theoretically. We showed that the model has two equilibrium states, which are disease-free and endemic equilibrium. The stability analyses show that the two equilibria states are locally and globally asymptotically stable theoretically, which are confirmed numerically using epidemiological data of COVID-19 pandemic. From our study, we observe that when $\kappa$ and $\beta$ decrease, the infected population also decreases. The biological implication of this is that the direct and latent infections are detrimental to the COVID-19 pandemic. Therefore, isolating exposed and visibly infected individual is an important strategy in controlling the COVID-19 pandemic.

## Data Availability

Authors can confirm that all relevant data sources are included in the article.

## Abbreviations

MERS: Middle East respiratory syndrome
SARS: Severe acute respiratory syndrome
WHO: World Health Organization
SEIR: Susceptible–exposed–infectious–recovered
DFE: Disease-free equilibrium
EE: Endemic equilibrium
GAS: Global asymptotic stability
LAS: Locally asymptotically stable
